# Characteristics of smell and taste disorders depending on etiology: a retrospective study

**DOI:** 10.1007/s00405-023-07967-1

**Published:** 2023-05-09

**Authors:** Mihnea Cristian Trache, Josef Maria Heinrich Schipp, Mareike Haack, Christine Adderson-Kisser, Catalina Högerle, Sven Becker, Christian Stephan Betz

**Affiliations:** 1 Department of Otorhinolaryngology, University Medical Center Hamburg-Eppendorf, Martinistraße 52, 20246 Hamburg, Germany; 2 Department of Otorhinolaryngology, Ludwig-Maximillian University Hospital (LMU), Munich, Germany; 3 Department of Otorhinolaryngology, University Hospital Tübingen, Tübingen, Germany

**Keywords:** Smell and taste disorders, Chemosensation, Rhinology, Olfactometry, Gustometry

## Abstract

**Purpose:**

This study investigates the impact of etiology on the epidemiologic profile, disease severity, type of treatment and therapy outcome in smell and taste disorders.

**Methods:**

This is a retrospective analysis of 270 patients that presented with a smell or taste disorder in a specialized, tertiary care center. An established questionnaire was used to collect data from patients and physicians. Olfactometry was performed with the Sniffin’ Sticks test kit, while gustometry was performed by taste strips.

**Results:**

Post-traumatic etiology was associated with young age (median 46 years) and male sex, and showed the most severe degrees of smell loss compared to other etiologies (64.3% anosmia). Postinfectious causes occurred more frequently in females (77.3%) and correlated with a history of pharyngeal surgery, suggesting a vulnerability for virally mediated sensory dysfunction following adenoid/tonsil removal. Parosmia also correlated with both postinfectious etiology (62.5%) and female sex. In sinunasal etiology, the presence of nasal polyps worsened the overall olfactory test score by approximately 50%. In particular, smell threshold and discrimination were reduced, while smell identification was not significantly impacted by nasal polyp obstruction. Sinunasal dysfunction was the only etiology to show significant improvement after therapy (73.9% improved). Finally, we could establish good correlations between the subjective impairment and objective dysfunction for each sensory modality.

**Conclusion:**

Each etiology of chemosensory dysfunction shows particular distributions of variables like sex, age, comorbidities and operations, disease severity, sensory threshold, discrimination and identification. This paper offers a detailed account of the correlations between the cause and the characteristics of smell and taste loss.

**Supplementary Information:**

The online version contains supplementary material available at 10.1007/s00405-023-07967-1.

## Introduction

Smell and taste function enables us to enjoy food, detect dangers and augments our non-verbal ways of communication and social interaction. Loss of smell and taste impairs quality of life by causing difficulties not only with eating, but also with personal hygiene and social relations and is, therefore, associated with depressive symptoms and loneliness [1]. Olfactory impairment makes one more likely to experience hazardous events [2] and low smell test scores may even predict a higher mortality rate in elderly patients [3].

The pathophysiology of smell and taste disorders is complex and can affect each layer of the sensory pathway. One differentiates between conductive causes, where there is an obstruction in the way to the sensory epithelium, as with septal deviation, mucosal swelling or nasal polyps, and sensorineural ones, such as viral infections and head trauma, that directly affect the smell receptors or taste buds, the afferent nerve fibers or central nervous structures involved in smell and taste perception. Sinunasal, postinfectious and post-traumatic etiologies together make up almost 90% of all smell disorders, while a variety of other causes account for the rest: idiopathic, iatrogenic, toxic, neoplastic or congenital [4]. The different pathomechanisms lead to different degrees of smell and/or taste dysfunction and subjective complaints and show different distributions of epidemiologic factors. The existing literature rarely distinguishes between different etiologies of smell and taste disorders and often focuses on either smell or taste, while comparisons between etiologies and correlations of the two chemical senses are scarce [5, 6]. The present work is a retrospective analysis of 270 patients that presented with a smell or taste disorder in a specialized center and investigates the prevalence and the distribution of epidemiological and clinical traits as well as detailed olfactometry and gustometry function in the different categories of smell loss, adding new data to the existing literature.

Smell and taste disorders affect a significant percentage of the general population. Hyposmia has a prevalence of about 15%, while approximately 5% of the population suffers from anosmia [7]. Gustatory sense is less frequently affected: only about 3% of patients that sought consultation in a medical smell and taste center showed a pathological gustometry result [8]. The multiple innervation of the taste mucosa with fibers from the seventh, ninth and tenth cranial nerves assures the robustness of the gustatory system compared to smell. A much higher percentage (66%) complained about a subjective loss of taste, and a large majority of those (87%) also complained about concurrent smell loss. Since the subjective taste is constructed from olfactory and gustatory afferent inputs, the impaired olfactory branch may be often “mistaken” for a taste dysfunction and reported as such by patients [8]. We help clarify the relationship between subjective impairment and sensory loss by comparing subjective questionnaire data to olfactometry and gustometry results.

The therapeutic approach and prognosis of smell and taste disorders are highly variable, naturally depending on the etiology. Conservative therapy employs corticosteroids, antibiotics, vitamins and and minerals as well as functional rehabilitation by olfactory training, while surgery mainly aims at resolving the obstruction towards the olfactory cleft. In the most prevalent, sinunasal hyposmia, sufficient therapy of the primary disease also alleviates loss of smell in 50–100% of patients [9, 10]. Postinfectious smell disorders can resolve spontaneously and respond to olfactory training in 25–71% of cases [11]. The less frequent, post-traumatic etiologies lack efficient causal therapies and show a poorer prognosis. We show data pertaining to therapy outcome in these separate etiologies.

This study aims at verifying known correlations and establishing new interdependencies between cause (etiology), clinical appearance and epidemiologic variables in chemosensory dysfunction.

## Methods

### Study design, patient cohort and data acquisition

In this retrospective study, anonymized data sheet analysis was performed on 280 patients who presented to the Department of Otorhinolaryngology at LMU Munich due to an olfactory or gustatory disorder in a specialized outpatient clinic over 7 consecutive years. Due to missing data, *n* = 10 patients were excluded. Socioeconomic variables such as age and gender, other secondary diagnoses, previously known allergies, previous operations, medication and a history of substance abuse were self-reported by the patients and collected by means of a defined questionnaire. This also included a detailed evaluation of the severity clinical course of olfactory or gustatory impairment. In addition, objective data was acquired by the physician in the second part of the questionnaire. This included a record of the type of therapy performed as well as olfactory and gustatory testing. The etiology of taste and smell disorder was determined by the physician. The questionnaire employed in this study had been previously developed by Temmel et al. in collaboration with the Working Group "Olfactology and Gustology" of the German ENT Society [12] (translation provided in supplementary information). In total, more than 30 variables were analyzed.

### Smell testing

Smell testing were performed using the Sniffin’ Sticks testing kit (https://www.burghart-mt.de/de/medizintechnik/sniffin-sticks-taste-strips.html) following the protocol described by Hummel et al. [13]. Odor threshold (T—the concentration where an odor is recognized), discrimination (D—the ability to tell two similar smells apart) and identification (I—the ability to name an everyday smell that is presented) were also examined. The subjects’ score range between 0 and 16 in each category. The results are entered and evaluated in a standardized manner using the publicly available software Olaf from the University Hospital Dresden (https://www.uniklinikum-dresden.de/de/das-klinikum/kliniken-polikliniken-institute/hno/forschung/interdisziplinaeres-zentrum-fuer-riechen-und-schmecken/neuigkeiten/downloads#software-downloads). The TDI score summed from the three subtests provides information about the subject's olfactory performance and ranged between 0 and 48. Olfactory dysfunction is classified into two severity levels: hyposmia (TDI 16.5–30.5) and functional anosmia (TDI < 16.5) while values above 30.5 are defined as normosmia [13].

### Taste testing

The screening taste test for assessing the taste qualities sweet, sour, bitter and salty is carried out with taste strips ("Taste-Strips") from the German company Burghart-Medizintechnik, Wedel as established by Mueller et al. [14]. The taste test participants had to choose each time between sweet, sour, salty and bitter in a forced-choice manner. There was only one taste strip per quality offered in an above-threshold concentration (the highest of four concentrations produced by the manufacturer). Between presentations of the different strips, the mouth is rinsed with water. Since the "bitter" taste was present the longest, it was offered last. A pathologic result was defined as failing to recognize at least one taste quality.

### Statistical analysis

Statistical analysis was performed using the SPSS statistical program (version 25). Data from the SPSS table were randomly checked for correctness with the original documents (questionnaire, patient file, test results). Categorical variables were reported in terms of absolute numbers (*n*) and percentage (%) and comparisons of proportions of a categorical variable in different groups were determined using Pearson's chi-squared test (if the frequency of the variable were greater than 5% in all groups) or Fisher's exact test (if at least one group showed a frequency of the variable less than 5%). Metric variables were expressed as median and their variation was expressed using the 25th and 75th percentiles, since normal distribution could not be assumed after the Shapiro–Wilk test. For group comparisons, the Mann–Whitney *U* nonparametric test for independent samples was chosen. Post-hoc adjustments using Bonferroni–Holm method were applied for multiple comparisons to counteract the cumulation of type I errors. For all tests, a two-sided *p* value < 0.05 was considered statistically significant. Box plots are used to visualize data (Table 1).

**Table 1:** Gender-specific distribution of main etiologies

Etiology	Total (*N* = 267)	Men (*N* = 115)	Women (*N* = 152)	*p *value
Post-traumatic	42 (15.7)	24 (20.9) *	18 (11.8)	*0.049*^*c*^
Postinfectious	75 (28.1)	17 (14.8)	58 (38.2) *	*0.000*^*c*^
Sinunasal	62 (23.2)	30 (26.1)	32 (21.1)	*0.383*^*c*^
Other	88 (32.9)	44(38.3)	44 (28.9)	*0.108*^*b*^
Idiopathic	12 (4.5)	8 (7.0)	4 (2.6)	*0.135*^*b*^
Toxic	9 (3.4)	5 (4.3)	4 (2.6)	*0.750*^*b*^
Congenital	7 (2.6)	4 (3.5)	3 (2.0)	*0.471*^*b*^
Neurodegenerative	5 (1.9)	1 (0.9)	4 (2.6)	*0.701*^*b*^
Other	55 (20.6)	26 (22.6)	29 (19.1)	*0.466*^*b*^

Frequency of different etiologies in smell and taste disorders

Percentage of column subgroup (sex) is shown in brackets. *p* value refers to comparison between genders, * significant correlation (*p* < 0.05) in ^b^ Fisher’s exact test or ^c^ Pearson’s chi-square test

## Results

The most common cause of dysfunction was the postinfectious (28.1%), followed by the sinunasal (23.2%) etiology. Postinfectious disorders showed a significantly more frequent occurrence in women (38.2% vs. 14.8%, *p* < 0.001). Conversely, a post-traumatic etiology (15.7% of cases) was diagnosed more often in men (20.9% vs. 11.8%, *p* = 0.049), while for the other etiologies the gender distribution was balanced (*p* > 0.05). Because of the low numbers of cases, the etiologies idiopathic, toxic, congenital and neurodegenerative as well as those which could did not fit into these groups were conflated to the category “other” for the further analyses, rounding up a total of 33% of patients.

**Fig. 1:**
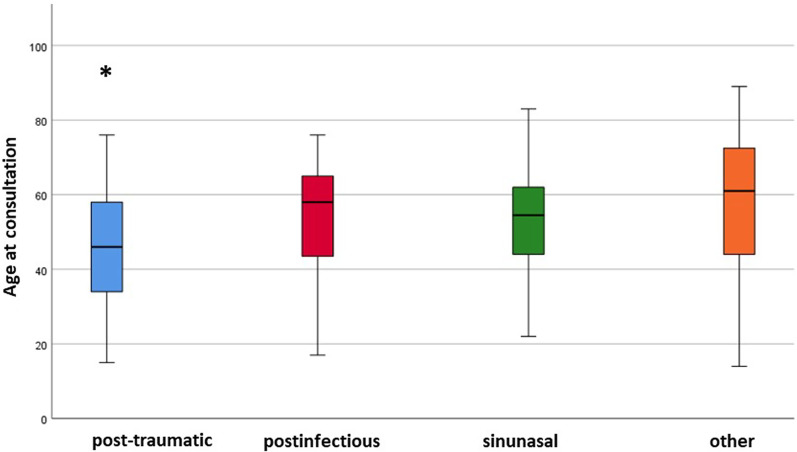
Age distribution in different etiologies. Box plots show median age, 25th and 75th percentile (box) and range (whiskers). *Significant difference (*p* < 0.05) between post-traumatic and every other etiology (Mann–Whitney *U* test with Bonferroni correction)

Since advanced age is known to correlate to olfactory decline, the patients’ age in different etiologies was plotted in Fig. 1.

Overall, the median age was 55 for men and 56 for women. Post-traumatic patients (median age 46) were significantly younger than each of the remaining groups.

### Etiology-dependent distribution of main variables

Different causes of taste and smell disorders can lead to different clinical presentations. Table 2 shows an overview of the most significant variables with respect to medical history, subjective complaints and objective findings in relation to the four main etiologies.

**Table 2:** Distribution of main variables across different etiologies

	Total *N*	Post-traumatic *n* (%)	Postinfectious *n* (%)		Sinunasal *n* (%)	Other *n* (%)
**Comorbidities**	**162**					
Diabetes	14 *0,473*^*b*^	1 (2.3) *0.703*^*b*^	3 (4.0)		4(6.4) *0.480*^*b*^	6 (6.8) *1.000*^*b*^
Hypertension	69 *0.324*^*b*^	10 (23.8) *0.764*^*b*^	16 (21.3) *0.354*^*b*^		16 (25.8) *0.075*^*b*^	29 (32.9) *0.823*^*b*^
Neoplasia	15 *0.061*^*b*^	1 (2.3) *0.533*^*b*^	1 (1.3) *1.000*^*b*^		2 (3.2) *0.532*^*b*^	12 (13.6)* *0.809*^*b*^
Reflux	27	4 (9.5)	8 (10.6)		5 (8.1)	11 (12.5)
Hypothyreosis	37	4(9.5)	13(17.3)		4 (6.4)	15 (17.0)
**Taste test results**	**214**					
Normogeusia	178	23 (71.9)	59 (95.2) *		39 (62.9)	57 (76.0) *
Hypogeusia	36	9 (28.1)	3 (4.8)		6 (9.7)	18 (24.0)
Comorbidities in Patients with Hpogeusia^**a**^:	29	Diabetes 2 (5.5)	Hypertension 15 (41.7) *	Neoplasia 3 (8.3)	Reflux 4 (11.1)	Hypothyreosis 5 (13.8)
**Smell test results**	**270**					
Normosmia	35	0 (0.0)	10(13.3)		10 (16.1)	15 (17.0)
Hyposmia	120 0	15 (35.7)	41 (54.7)		29 (46.8)	34 (38.6)
Anosmia	115	27 (64.3) * **0.667**^**c**^	24 (32.0)		23 (37.1) 0.212^c^	39 (44.3)
**Parosmia**^**b**^	**48**	6 (12.5)	30 (62.5) * ^**b**^		3 (6.2)	9(18.8)
**Subjective impairment**	**268**					
Extreme	88	22 (53.7) *	27 (36.0)		18 (30.0)	21 (22.8)
Strong	106	10 (24.4)	31 (41.3)		23 (38.3)	42 (45.6)
Moderate	66	9 (21.9)	17 (22.7)		16 (26.7)	24 (26.1)
Mild	8	0 (0.0)	0 (0.0)		3 (5.)	5 (5.4)
**Surgery**	**109**					
Nose/sinus	36	2 (16.6)	3 (12.5)		17 (54.8) *	14 (33.3)
Oral/pharynx	58	8 (66.7)	21 (87.5) *		7 (22.6)	22 (52.4)
Ear	1	1 (8.3)	0 (0.0)		0 (0.0)	0 (0.0)
Several	14	1 (8.3)	0 (0.0)		7 (22.6)	6 (14.3)
**Therapy**	**231**					
Primary therapy	142	31 (88.6) *	57 (80.3) *		22 (40.7)	32 (45.1)
Extended therapy	89	4 (11.4)	14 (19.7)		32 (59.3) *	39 (54.9) *
**Therapy outcome**	**71**					
Improvement	32	2 (16.7)	7(50.0)		17 (73.9) *	6 (25.0)
No improvement	39	10 (83.3)	7 (50.0)		6 (26.1)	18 (75.0)

Percentage of patients with a specific etiology is shown in brackets (i.e. 95.2% of postinfectious patients that underwent gustometry suffered from hypogeusia); ^a^percentage of total hypogeusia patients (36); ^b^percentage of total patients that report parosmia (48). *Significant correlation (*p* < 0.05) in Fisher’s exact test or Pearson’s chi-square test (see Materials and methods)

We found no associations between comorbidities and the specific etiologies post-traumatic, postinfectious and sinunasal, while the category “other” correlated significantly with history of neoplasia (80% of neoplasia patients classified as “other”).

Although a majority of patients complained about taste or fine taste (Table 3), pathologic taste test results were rare (16.8% hypogeusia). Men were significantly more likely to have a quantifiable taste dysfunction than women (18.8% vs 9.2%, *p* = 0.029). Hypogeusia patients were also more likely to have a history of arterial hypertension (41.7% in hypogeusia vs. 21.9% in normogeusia, *p* = 0.017). The other comorbidities, including reflux disease, were not associated with taste disorders.

**Table 3:** Subjective main problem in relation to taste and smell teste results

	Smell	Fine taste	Taste
Total (*N* = 270)	241 (89.3%)	162 (60.0%)	56 (20.7%)
Men (*n* = 117)	103 (88.0%)	70 (60.0%)	27 (23.1%)
Women (*n* = 153)	138 (90.2%)	92 (60.1%)	29 (19%)
Normogeusia (*n* = 178)	160 (89.9%)	112 (62.9%)	26 (14.6%)
Hypogeusia (*n* = 36) Men: 22 (18.8%) * Women: 14 (9.2%)	31 (86.1%)	20 (55.5%)	14 (38.9%) *
Normosmia (*n* = 35)	27 (77.1%)	21 (60.0%)	6 (17.1%)
Hyposmia (*n* = 120)	104 (86.7%)	68 (56.7%)	27 (22.5%)
Anosmia (*n* = 115)	111 (96.5%) *	73 (63.5%)	23 (20.0%)

Percentage of row subgroup (sex/taste test/smell test) is shown in brackets. *Significant correlation (p < 0.05) in Pearson’s chi-square test

With regard to the severity of the smell disorder, post-traumatic patients were the most affected and the only group significantly more likely to lose the sense of smell entirely (64.3% anosmia, *p* = 0.002), while postinfectious and rhinosinusitis patients tended to suffer more from hyposmia than from anosmia (*p* > 0.05). The degree of subjective impairment aligned with the severity of sensory loss and was significantly higher in post-traumatic patients.

Parosmia was more prevalent in women than men (*p* = 0.015) and found to strongly correlate with a postinfectious genesis—two thirds of patients reporting parosmia suffered from a postinfectious etiology.

With respect of surgical history, sinunasal patients are naturally more prone to nose and sinus operations, while postinfectious etiology correlated with history of oropharyngeal surgery.

The therapy performed as well as the therapeutical outcome were finally surveyed. Post-traumatic and postinfectious were most likely to receive standard therapy (*p* < 0.01), whereas sinunasal and other etiologies were more often treated by extended therapy (*p* < 0.01). Data regarding outcome of therapy were only available for 71 (26.3%) of patients. Only the sinunasal etiology was significantly more likely to show improvement after therapy (27.4% show improvement vs. 9.6% show no improvement).

### Correlation of subjective complaints with sensory loss

To further investigate the relationship between subjective complaints and sensory function, the subjective main problem was surveyed and put in relation to the taste and smell test results (Table 3). Patients had to choose between impairment of smell, fine taste (such as aromas) or taste (sweet, sour, salty, bitter) as main problem. Multiple answers were permitted.

Most patients (89.3%) complained about smell loss as the main problem, while 60% complained of fine taste and only a minority (20.7%) of strict taste impairment. Patients who reported strict taste impairment as the main problem were also more likely to have a pathologic taste test result (*p* < 0.01). Similarly, those reporting smell loss as the main problem were more likely to suffer from anosmia rather than hyposmia or normosmia (*p* < 0.01). Subjective impairment of fine taste was present in a majority of patients regardless of their olfactometry/gustometry results.

### Smell threshold, discrimination and identification patterns in different etiologies

**Fig. 2:**
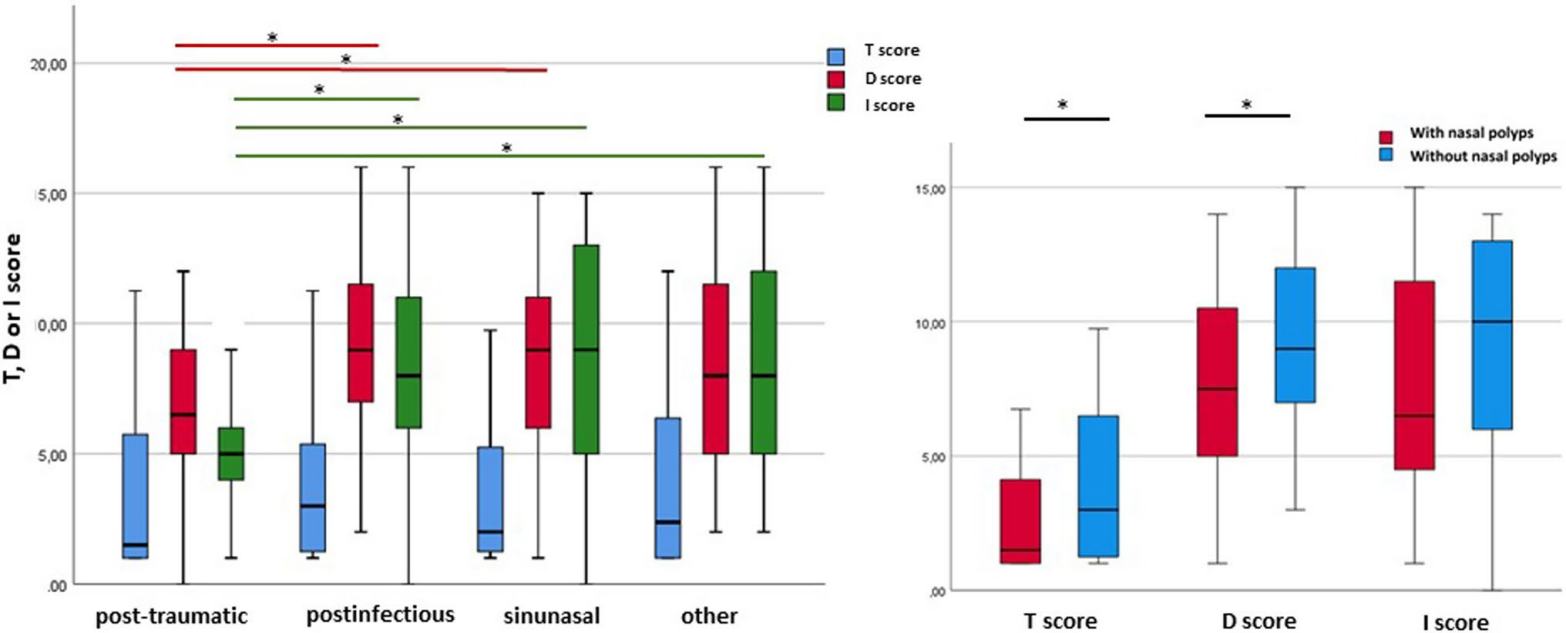
Smell threshold, discrimination and identification. Left: threshold (blue), discrimination (red) and identification (green) scores in the main etiologies. *Significant differences (*p* < 0.05) in D and I score between post-traumatic and other etiology groups (Mann–Whitney *U* test). Right: threshold, discrimination and identification scores with and without nasal polyps. *Significant differences (*p* < 0.05) in T or D score between nasal polyps and no nasal polyps (Mann–Whitney *U* test)

Post-traumatic smell loss shows significantly worse smell discrimination and identification than other etiologies but no significant differences regarding smell threshold. To identify the effect of nasal blockage by polyposis nasi on smell dysfunction we compared patients with nasal polyps against the other patients suffering from sinunasal smell loss. Taken individually, threshold and discrimination of smell were significantly worse in sinunasal patients with nasal polyps than in ones without (Fig. 2, right), while the combined TDI score was nearly two times lower in nasal polyps patients. Smell identification ability was not significantly influenced by nasal polyps.

## Discussion

### Purpose of this study

This study examines smell and taste disorders of different etiologies with respect to their clinical appearance. This includes quantitative sensory loss as well as smell threshold, discrimination and identification data, subjective complaints, medical history, clinical course, therapy and outcome. This data enables us to delineate a clinical profile for each etiology (see below). Of course, the inherent limitations of a retrospective single-center questionnaire study apply here.

### Clinical appearance of different causes of smell and taste loss

The classification of cases in different etiological groups may itself be problematic, since the physician decides upon the etiology mainly based on the patient’s history. For instance, assignment to a postinfectious etiology requires a temporal association between smell/taste loss and an upper airway infection, but the causality cannot be verified and sometimes the cause may lie elsewhere. In neurodegenerative diseases, for example, smell loss can occur years before the neurological symptoms that ultimately lead to diagnosis.

The sex distribution was even in the entire study cohort. Postinfectious and post-traumatic causes distributed unevenly between male and female sex with a significantly higher likelihood of females to suffer from postinfectious, and of men to suffer from post-traumatic taste loss. While the latter is explained by the “risk appetite” of the male sex [15], the connection between female sex and postinfectious smell loss has not yet been elucidated. Females are also more likely to suffer from parosmia, as are patients suffering from postinfectious smell loss. The clustering of these three variables raises the question of a confounder, which cannot be clarified in our study. While it is known that females outperform males in olfactory tests, it is unclear why they seem to suffer more than males from postinfectious olfaction impairment.

Post-traumatic chemosensory disorders impact the smell and taste function most severely. Unlike in the other categories, a majority of post-traumatic patients were anosmic and reported extreme subjective impairment, despite their significantly younger age. This is in line with previous literature [16]. The high disease severity does not necessarily correlate with extensive therapeutic efforts, since a large majority of post-traumatic patients only received primary therapy. Direct traumatic disruption of the olfactory pathway is arguably irreversible in most cases, and there is still a lack of appropriate treatment to influence this type of sensorineural smell loss.

Postinfectious cases are described by a sudden onset (84%) and severe subjective dysfunction: more than three quarters complain about either extreme or strong impairment. The majority of postinfectious patients were hyposmic (55%) and a considerable amount (32%) were anosmic. However, taste loss was observed less frequently in postinfectious patients compared to the other etiologies, which is in disagreement with previous literature [16]. Interestingly, there was a significant association between postinfectious disease and history of operations in the pharynx, especially tonsil and adenoid removals. It might be interesting to investigate whether adenotonsillectomy might lead to a weakened immune defense against the viral pathogen, facilitating infection and dysfunction of the sensory epithelium. Finally, most postinfectious patients received primary therapy. Indeed, the good therapeutic response to olfactory training is well known in postinfectious smell loss, while corticosteroids—local or systemic—show no additional benefit [17]. We could not identify any improvement after therapy in postinfectious cases. Notably, data to therapy outcome was scarce (26.3%).

Sinunasal patients are predominantly hyposmic (46.8%) and report strong complaints. The nasal polyps subgroup scored worse than other sinunasal patients in the smell test. This is well known and surely one of the reasons for the generous interest in the recent advances in antibody therapy for nasal polyps [18]. Moreover, smell loss is a main criterium for recommending antibody therapy [19]. In contrast to the other etiologies, sinunasal disease primarily impacts smell conduction, making it more responsive to therapies that “clear up” the nose. This is the only subgroup to report an improvement of symptoms following therapy, despite the scarce data available for disease outcome. Documenting and quantifying therapeutic response is often overlooked in the clinical routine and patients often receive continuous treatment without significant effect. Gathering outcome data is essential for filtering out effective treatments and avoiding unnecessary ones.

### Smell threshold, discrimination and identification

There are only little data available on smell threshold, discrimination and identification function in different etiologies [16]. This study shows that mechanical damage to the olfactory filaments or other central structures, like in post-traumatic etiology, lead to a significantly reduced ability to tell smells apart (discrimination) and identify known smells (identification) compared to the other etiologies. On the other hand, when there is a conductive problem as with nasal polyps, smell threshold and discrimination are significantly impaired. While nasal blockage might lead to a decreased odor concentration around the olfactory mucosa and thus explain the change of threshold, further work is needed to clarify the exact connection between sensorineural damage and higher olfactory functions such as discrimination and identification.

### Parosmia

The presence of parosmia strongly correlates with a postinfectious etiology. The mechanism of parosmia supposes a mis-wiring of olfactory neurons to the glomerulus during regeneration and is associated with olfactory recovery (peripheral mis-wiring theory) [20, 21]. This is coherent with the pathophysiology of postinfectious smell loss [22, 23]. Interestingly, in our study cohort, women are significantly more likely to suffer from parosmia than men, which is in line with previous findings [24]. The influence of sex on the development and perception of parosmia is yet to be systematically addressed in a systematic manner.

### Taste impairment: subjective and objective

In our study, men were more frequently affected by hypogeusia than women. Previous reports on sex differences in gustatory function are divergent [25, 26] and the nature of these differences is still a matter of debate. Hypogeusia also correlated with history of arterial hypertension. It is known that patients under beta blocker, ACE-inhibitor or calcium antagonist therapy have lower electrogustometric thresholds than healthy people [27, 28]. Our result may be interpreted as a side effect of antihypertensive drug intake.

Taste testing was performed using one taste strip per taste quality in an above-threshold concentration. Although this method maximizes sensitivity, it is a limitation of this study by increasing the chance for false positive results. The low prevalence of true gustatory disorders is well known (13–20%) [29]. Complaints of taste loss, in comparison, are much more frequent than objective dysfunction and are thought to reflect rather the loss of smell, not taste, function [8]. We show that, when allowing patients to differentiate between fine taste and taste in their reports, there is a correspondence between complaints and sensory impairment. Complaints of strict taste impairment are present in a similar amount of patients as those with a pathologic taste test result and the two variables correlated significantly. Similarly, anosmia correlated with reporting smell loss as main problem. Reporting fine taste disturbances was a prevalent (> 55%) but unspecific trait of both hyposmic/anosmic and hypogeusic patients, confirming that flavor perception results from the integration of these two different chemosensory channels.

## Supplementary Information

Below is the link to the electronic supplementary material.

Supplementary file1 (PDF 167 KB)

## References

[1] Boesveldt S, Postma EM, Boak D (2017). Anosmia-a clinical review. Chem Senses.

[2] Santos DV, Reiter ER, DiNardo LJ, Costanzo RM (2004). Hazardous events associated with impaired olfactory function. Arch Otolaryngol Head Neck Surg.

[3] Ekström I, Sjölund S, Nordin S (2017). Smell loss predicts mortality risk regardless of dementia conversion. J Am Geriatr Soc.

[4] Damm M, Temmel A, Welge-Lüssen A (2004). Olfactory dysfunctions. Epidemiology and therapy in Germany Austria and Switzerland. HNO.

[5] Stinton N, Atif MA, Barkat N, Doty RL (2010). Influence of smell loss on taste function. Behav Neurosci.

[6] Landis BN, Scheibe M, Weber C (2010). Chemosensory interaction: acquired olfactory impairment is associated with decreased taste function. J Neurol.

[7] Brämerson A, Johansson L, Ek L, Nordin S, Bende M (2004). Prevalence of olfactory dysfunction: the skövde population-based study. Laryngoscope.

[8] Deems DA, Doty RL, Settle RG (1991). Smell and taste disorders, a study of 750 patients from the university of Pennsylvania smell and taste center. Arch Otolaryngol - Head Neck Surg.

[9] Damm M, Jungehülsing M, Eckel HE, Schmidt M, Theissen P (1999). Effects of systemic steroid treatment in chronic polypoid rhinosinusitis evaluated with magnetic resonance imaging. Otolaryngol Neck Surg.

[10] Heilmann S, Huettenbrink KB, Hummel T (2004). Local and systemic administration of corticosteroids in the treatment of olfactory loss. Am J Rhinol.

[11] Hummel T, Rissom K, Reden J, Hähner A, Weidenbecher M, Hüttenbrink KB (2009). Effects of olfactory training in patients with olfactory loss. Laryngoscope.

[12] Temmel AFP, Quint C, Schickinger-Fischer B, Klimek L, Stoller E, Hummel T (2002). Characteristics of olfactory disorders in relation to major causes of olfactory loss. Arch Otolaryngol Head Neck Surg.

[13] Hummel T, Kobal G, Gudziol H, Mackay-Sim A (2007). Normative data for the “Sniffin’ Sticks” including tests of odor identification, odor discrimination, and olfactory thresholds: an upgrade based on a group of more than 3,000 subjects. Eur Arch Oto-Rhino-Laryngol.

[14] Mueller C, Kallert S, Renner B (2003). Quantitative assessment of gustatory function in a clinical context using impregnated “taste strips”. Rhinology.

[15] Hayakawa H, Fischbeck PS, Fischhoff B (2000). Traffic accident statistics and risk perceptions in Japan and the United States. Accid Anal Prev.

[16] Migneault-Bouchard C, Hsieh JW, Hugentobler M, Frasnelli J, Landis BN (2020). Chemosensory decrease in different forms of olfactory dysfunction. J Neurol.

[17] Genetzaki S, Tsakiropoulou E, Nikolaidis V, Markou K, Konstantinidis I (2021). Postinfectious olfactory dysfunction: oral steroids and olfactory training versus olfactory training alone: is there any benefit from steroids?. ORL.

[18] Patel GB, Peters AT (2021). The role of biologics in chronic rhinosinusitis with nasal polyps. Ear Nose Throat J.

[19] Fokkens WJ, Lund VJ, Hopkins C (2020). European position paper on rhinosinusitis and nasal polyps 2020. Rhinology.

[20] Parker JK, Kelly CE, Gane SB (2022). Insights into the molecular triggers of parosmia based on gas chromatography olfactometry. Commun Med.

[21] Liu DT, Sabha M, Damm M (2021). Parosmia is associated with relevant olfactory recovery after olfactory training. Laryngoscope.

[22] Reyna RA, Kishimoto-Urata M, Urata S, Makishima T, Paessler S, Maruyama J (2022). Recovery of anosmia in hamsters infected with SARS-CoV-2 is correlated with repair of the olfactory epithelium. Sci Rep.

[23] Rombaux P, Mouraux A, Bertrand B, Nicolas G, Duprez T, Hummel T (2006). Olfactory function and olfactory bulb volume in patients with postinfectious olfactory loss. Laryngoscope.

[24] Rashid RA, Alaqeedy AA, Al-Ani RM (2021). Parosmia due to COVID-19 disease: a 268 case series. Indian J Otolaryngol Head Neck Surg Off Publ Assoc Otolaryngol India..

[25] Hans M, Hans VM, Kahlon N, Sagar M, Pandey AK, Das A (2022). Gustatory dysfunction and oral ulceration in COVID-19 patients: a cross sectional study. Dent Res J.

[26] Wang JJ, Liang KL, Lin WJ, Chen CY, Jiang RS (2020). Influence of age and sex on taste function of healthy subjects. PLoS ONE.

[27] Suliburska J, Duda G, Pupek-Musialik D (2012). The influence of hypotensive drugs on the taste sensitivity in patients with primary hypertension. Acta Pol Pharm.

[28] Ackerman BH, Kasbekar N (1997). Disturbances of taste and smell induced by drugs. Pharmacotherapy.

[29] Pribitkin E, Rosenthal MD, Cowart BJ (2003). Prevalence and causes of severe taste loss in a chemosensory clinic population. Ann Otol Rhinol Laryngol.

