# Supplementary material for: Characteristics of smell and taste disorders depending on etiology: a retrospective study
Source: Eur Arch Otorhinolaryngol. 2023 May 9;280(9):4111–9. doi: 10.1007/s00405-023-07967-1 (PMC10382332; doi:10.1007/s00405-023-07967-1)
Supplement: Supplementary file 1 — Supplementary file1 (PDF 167 KB) [file 405_2023_7967_MOESM1_ESM.pdf]

Smell / Taste Questionnaire

Date:

Treating physician:

Personal Information

Tel. nr. (private):

Tel. nr. (work):

To be filled in by the patient

What does the main problem concern?<br>(multiple answers are allowed)	☐ smell<br>☐ fine taste<br>☐ taste (sweet, sour, salty, bitter)
For how long has the problem persisted?	☐ less than 3 months ☐ between 3 and 24 months ago<br>☐ more than two years ☐since I can remember<br>☐ don't know
How did the problem begin?	☐ gradually ☐ suddenly<br>☐ since birth ☐ don't know
How has the problem changed so far?	☐ has gotten better<br>☐ has not changed<br>☐ has gotten worse
What do you consider the cause of the problem?	☐ accident ☐ cold / infection<br>☐ drug / medicine intake ☐ operation<br>☐ mouth dryness ☐ denture<br>☐ polyps ☐ sinus inflammation<br>☐ other (please specify): ____________________
Do you suffer from a chronic disease involving the nose or the sinuses?<br>Please select if applicable	☐ no ☐ running nose<br>☐ yes ☐ sneezing<br>☐ polyps<br>☐ recurrent sinus inflammation (more than 3x/year)
Do you have allergies?	☐ no (negative allergy test)<br>☐ no symptoms (never tested)<br>☐ yes (please specity):____________________
Is your impairment variable or constant?	☐ variable<br>☐ constant<br>☐ don't know<br>☐ influenced by certain circumstances: ____________________
How strongly are you impaired by the smell/taste problem?	☐ extremely ☐ strongly ☐ moderately<br>☐ mildly ☐ not at all

Please fill in following table in the case of taste impairment

The taste impairment refers to the perception of which taste?	☐ sweet ☐ sour ☐ salty<br>☐ bitter ☐ spicy ☐ none of these
Do you experience following sensations durably in your mouth?	- burning ☐ yes ☐ no<br>- bitter taste ☐ yes ☐ no<br>- salty taste ☐ yes ☐ no<br>- sour taste ☐ yes ☐ no<br>- dry mouth ☐ yes ☐ no<br>- foreign body sensation ☐ yes ☐ no

Smell / Taste Questionnaire

Date:

Treating physician:

Personal Information

Tel. nr. (private):

Te. nr. (work):

To be filled in by the physician

Weight loss due to smell/taste disorder	☐ no<br>☐ yes: _____kg / _____ years
Long-term medication? Please specify	☐ none<br>☐ yes: ____
Chronic diseases? Please specify	☐ no ☐ yes<br>☐ diabetes mellitus<br>☐ hypertension<br>☐ neoplasia<br>☐ other: ___________________
Operations in the head region	☐ sinus surgery ☐ septum surgery<br>☐ nasal polyp surgery ☐ turbinate surgery<br>☐ tonsil surgery ☐ adenoid surgery<br>☐ middle ear surgery ☐ dental surgery<br>☐ dental surgery ☐ other: ______________<br>**Side** ☐ left ☐ right
Flu vaccination	☐ no ☐ yes<br>(evtl. timepoint: ______________)
Smoking history	☐ no ☐ yes: ______ PY
Alcohol	☐ no ☐ occasionally ☐ regularly
For idiopathic smell/taste disorder:<br>Family history of:	Alzheimer's disease: ☐ no ☐ yes<br>Parkinson's ☐ no ☐ yes
Parosmia ☐ no ☐ yes<br>☐ left side ☐ right side	☐ daily ☐ not daily<br>☐ very intense ☐ moderate<br>☐causes weight loss ☐ does not cause weight loss

Test results

Sniffin' Sticks **T:**………… ; **D:** …………; **I:** …………;

Taste test (4 Sprays): ……………………….;

Clinical findings:

Septum deviation: ☐ to the left ☐ to the right ☐ no deviation

Olfactory cleft visible: ☐ left ☐ right

Polyposis nasi: left: ☐ I° ☐ II° ☐ III°
right: ☐ I° ☐ II° ☐ III°

Suspected etiology: ☐ posttraumatic ☐ toxic ☐ postinfectious ☐ congenital
☐ sinunasal ☐ neurodegenerative ☐ idiopathic ☐ other
